# Utilization of the fermentation residues of validamycin as a potential platform for seed treatment†

**DOI:** 10.1039/c7ra12298e

**Published:** 2018-03-09

**Authors:** Bo Zhang, Shaowei Liao, Tianrui Ren

**Affiliations:** The Key Laboratory of Resource Chemistry of Ministry of Education, The Development Centre of Plant Germplasm Resources, College of Life and Environmental Science, Shanghai Normal University 100 Guilin Road Shanghai 200234 P. R. China trren@shnu.edu.cn +86 21 64328850 +86 21 64328850

## Abstract

Seed coating is a versatile means to control crop pests and diseases, which can effectively reduce pesticide quantity and improve pesticide efficacy. Herein, 6% tebuconazole flowable concentrate for seed treatment (FS) was prepared by using the validamycin fermentation residue (VFR) as a pesticide carrier. Then, the effects of the VFR and dispersants on the physicochemical properties of the FS were systematically evaluated. Moreover, the germination rate and antibacterial performance of wheat seeds coated with FS were investigated through a bioactivity experiment. The experimental results show that the FS prepared by VFR, dispersant SD-816 and NNO exhibits superior suspension stability. The suspension rates at 1 h and 24 h are 99.78% and 95.11% respectively. Furthermore, the viscosity of the system only slightly changes after 30 days. In addition, the obtained FS system exhibits shear thinning behavior under high speed shearing showing typical features of pseudo-plastic non-Newtonian fluids, which conforms to the Herschel–Buckley model. A bioactivity experiment showed the germination rate of coated wheat seeds reached 90%, and increased by 7.0% and 3.0% compared to the control group and positive control Raxil respectively. Futhermore, the antibacterial rate reached 80.5%, and was higher than the control group and Raxil. In addition, the obtained FS was very efficacious in controlling wheat powdery mildew compared to the commercial formulation Raxil.

## Abstract 2

Validamycin fermentation residue (VFR) is used as a pesticide carrier for seed treatment (FS). FS coated wheat seeds have higher germination and antibacterial rates and better protection against wheat powdery mildew than those treated with Raxil.

## Graphical abstract

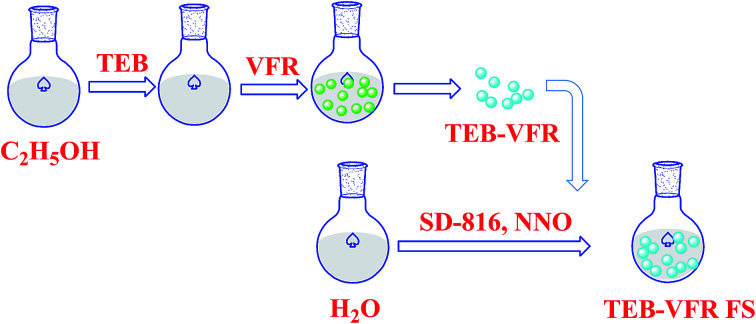

## Introduction

Seed coating technology is a precise application technology, which can improve the efficacy of pesticides and the performance and physical properties of seeds.^1,2^ Moreover, it can also promote germination and survival of the seed under adverse environmental conditions.^3–6^ Currently, seed coating technology has been widely applied in rice,^7^ sorghum,^8^ cotton,^9^ beans,^10–12^ corn,^13^ cereals,^14^ winter wheat,^15–17^ sugar-beet^18^ and various vegetable crops.^19–22^ Yang^23^ found that the corn seed was treated with 60 g L^−1^ tebuconazole FS, and when the dosage was 50–150 mL/100 kg, it can significantly improve the emergence of maize and promote the maize growth. In addition, its control efficiency against maize smut was 57.03–91.06%, and the corn yield was increased by 25.8% to 44.4%. Zeng^24^ have found that rice seeds after coating increased by 5% compared to traditional rice seeds.

Validamycin is an effective aminocyclitols antibiotic produced by fermentation of *Streptomyces hygroscopicus* var. *limoneus* or *S. hygroscopicus* var. *Jinggangensis*,^25^ and exhibits excellent therapeutic effects to rice sheath blight caused by *Rhizoctonia solani*.^26^ However, in its main production process, microbial fermentation produces a large number of solid fermentation residues, which results in serious waste of resources and made strong impacts on the environment. Amazingly, VFR contains a lot of useful elements, such as protein and carbohydrate, and shows good biocompatibility and environmental friendliness. Therefore, it is very essential to develop a versatile means of the comprehensive utilization of VFR.

To address this problem, VFR is explored as pesticide carriers to prepare the FS. Moreover, the suspension rate, viscosity and rheological properties of the obtained FS were systematically investigated. In addition, the bioactivity experiment were conduct to study its impacts on the germination of wheat seeds and control the powdery mildew of wheat. This study provides a good solution for the comprehensive utilization of FS.

## Experimental

### Materials

Technical grade tebuconazole (98.5% purity) was kindly supplied by Jiannong jiangsu Agrochemical & Chemical Co. Ltd. (China). Absolute ethanol (99.5%) was obtained from Sinopharm Chemical Regent Co. (China). The flowable suspension of tebuconazole (Raxil, 60 g L^−1^) was the gift of Bayer Crop Science AG. VFR was generously supplied by Zhejiang Tonglu Haifeng Biosciences Co., Ltd. (Zhejiang, China). Dispersant NNO (2-naphthalenesulfonic acid) and SD-816 (sodium polycarboxylic acid) were provided by Shanghai ShiDa Macromolecule Material Co (Shanghai, China). All chemicals were analytic grade and were used without further purification. Double distilled water was used in the experiment.

### Fabrication of TEB–VFR

VFR was hyperthermia inactivation, and spray dried at 220 °C. Then, 1.0 g of VFR were dispersed TEB ethanol solution (6.0 g of TEB was dissolved in absolute ethanol) under continuous shaking for 24 h at 30 °C. Finally, organic solvent was removed by rotary evaporation, and the obtained TEB–VFR was used to prepare the FS.

### Preparation of flowable concentrate for the seed coating agent

FS was prepared by wet sand grinding technology. The optimum formulation of FS was determined by pre-experiment. Typically, 7 g of TEB–VFR, 2 g of SD-816, 1 g of NNO, 0.5 g of xanthan gum, 4 g of ethylene glycol and 0.5 g of rhodamine B were mixed together with the rest of the water, and were ground by using a sand mill until the particle size distribution of less than 5 μm. And then the optimum formulation was obtained, which is used for seed treatment. The concentration of TEB in this FS was 6%.

### Determination of suspension rate^27^

5 g of the obtained FS was diluted to 250 mL with distilled water. Then, the suspension rate was determined at 30 °C after 1 h, 3 h, 6 h, 12 h, 24 h and 48 h, respectively. The samples were repeated 3 times.

### Determination of rheological properties

The rheological properties of the obtained FS was determined using Haake Mars III advanced modular. The relationships between shear rate and shear stress, as well as apparent viscosity of the system were investigated with shear rate varying from 0 to 100 s^−1^. The rheological curves were fitted with a Herschel–Bulkley model.

### Determination of viscosity of the obtained FS

The viscosity of seed coating agent was determined by NDJ-1 Rotary digital viscometer on 1st day and 30th day respectively. The viscosities of the samples were measured three times in parallel.

### Seed germination test

The FS and wheat seeds were added to the triangular bottle in a certain proportion. The mixture was shaken well in the shaker, placed on Petri dishes, and drying at 35 °C. The germination test of coated wheat seeds was conducted by Agar-agar bed^28^ in an incubator under controlled conditions (25–28 °C, photoperiod of 12 h, and 85% relative humidity). Each treatment repeated three times. Germination potential, germination rate, germination index and vigor index were investigated once a day.^29^ After 7 days of incubation, the seedling was determined the seedling quality. Uncoated seeds and Raxil seed coating agents were as control. The numbers of germinated seeds on the day 3 and 7 after initiation were germination energy and germination percentage, respectively. Germination index (GI) and vitality index (VI) were calculated following the equations:

1 GI = ∑(G_*t*_/D_*t*_)

2 VI = GI × *S*

where, G_*t*_ means germination rate at day *t*, D_*t*_ means day *t*, S means shoot length.

### Antibacterial test for FS

0.25 mL, 0.20 mL and 0.17 mL of self-made FS, as well as 0.2 mL of water and 0.2 mL of Raxil were added into 10 mL sterilized PDA culture medium, respectively. These mediums were poured into Petri dishes after being shaken. Then, *Fusarium graminearum* and powdery mildew were placed in the center of Petri plates, and incubated at 25 °C. After 72 h, their growth was observed to determine the inhibitory effect of various FS. Assays were carried out in triplicates.

### Bioassay experiments

The bioactivities of the obtained FS and commercial formulation of Raxil on wheat powdery mildew which is one of main wheat diseases were conducted on 2–3 leaf stage of wheat seedlings. Typically, 10 grain of wheat seeds coated with two FS were put into flowerpot with diameter 15 cm. Then 50 mL of bacteria solution of powdery mildew sprayed evenly on wheat seeds. Wheat seeds were grown in a greenhouse under conditions of 18 °C, at 70% relative humidity, with 12 h light and were used for investigating the emergence rate of wheat seeds and the control efficiency of powdery mildew after 7 and 12 days, respectively. Blanks uncoated seeds and three replicates of each sample were used for each series of experiments.

### Characterization

Surface morphologies of the samples were observed by field emission scanning electron microscopy (FESEM, 4800S, Hitachi, Japan). Surface chemical analyses of samples were performed by X-ray photoelectron spectroscopy (XPS) using a PHL1600ESCA instrument equipped with monochromatic Al Kα X-ray source operated at 250 W. The viscosities of the samples were measured by digital rotary viscosimeter (NDJ-1, Shanghai Hengping instrument, China). The rheological properties of the samples were studied by anton paar rheometry (MCR302, Austria).

## Results and discussion

**Scheme 1:**
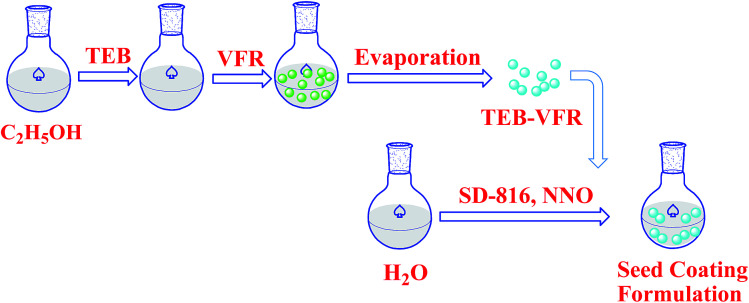
Schematic illustration of the preparation process for the TEB–VFR FS.

**Fig. 1:**
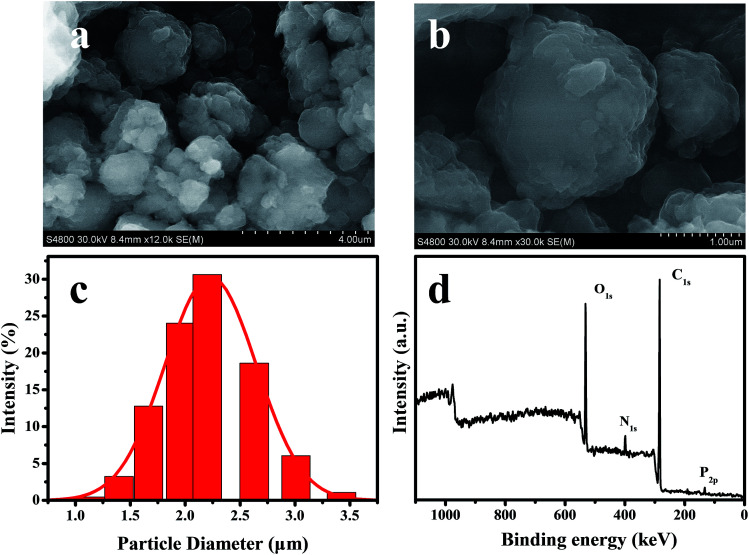
FESEM images of VFR (a and b), size distribution histogram of VFR (c), XPS full survey spectrum of VFR (d).

Scheme 1 schematically demonstrates the synthesis process of TEB–VFR FS. First, VFR was explored as pesticide carrier to prepare the TEB–VFR. As demonstrated in Fig. 1a and b, VFR had a rough surface, suggesting that it is beneficial to adsorb pesticide molecules. Moreover, the particle size of FS is an important factor to improve its stability. The particle size of well-qualified FS is less than 5 μm. Fig. 1b and c shows that VFR is quasi-spherical in shape with an average size of 2.23 μm (Fig. 1c), which is expected to fabricate the well-qualified and stable FS, and make FS evenly around the seed surface. Additionally, VFR contains abundant crude protein and starch. And our results (Fig. 1d) are further proved that VFR contains many useful elements, such as carbon, oxygen, nitrogen and phosphorus. Moreover, it is noteworthy that VFR has no other harmful elements such as heavy metals. Therefore, VFR may contribute to improve the seed germination. In the second step, the mixtures of TEB–VFR, water, dispersant SD-816 and NNO, as well as other additives were fully mixed and ground to obtain FS samples.

**Fig. 2:**
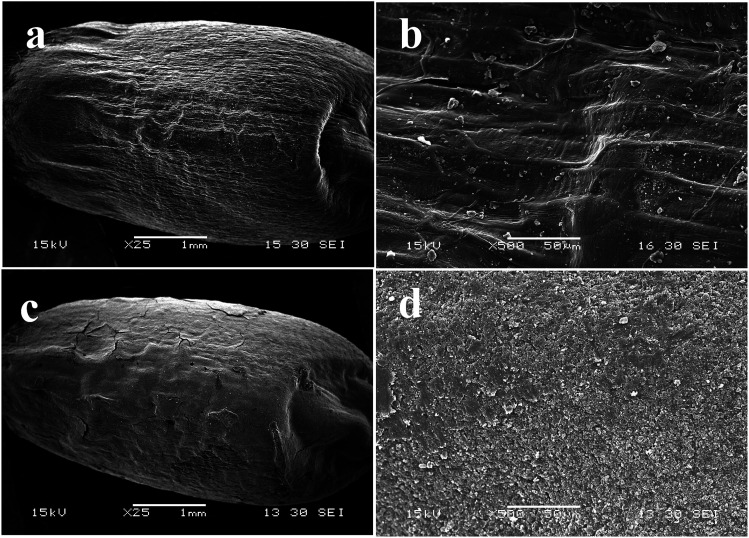
SEM images of wheat seed, before (a and b) and after (c and d) coated with TEB–VFR FS.

The SEM was employed to investigate the coating uniformity of TEB–VFR FS on wheat seeds surface. In comparison to the original wheat seeds (Fig. 2a and b), a thin layer covered the whole outer surface of wheat seed (Fig. 2c), indicating that the TEB–VFR FS has been successfully immobilized on wheat seed. Meanwhile, FS exhibited the superior coating uniformity on the surface of wheat seed (Fig. 2d), which actually proved that VFR could serve as an excellent platform to prepare FS used for seed treatment, and correlate well with the Fig. 1c.

**Fig. 3:**
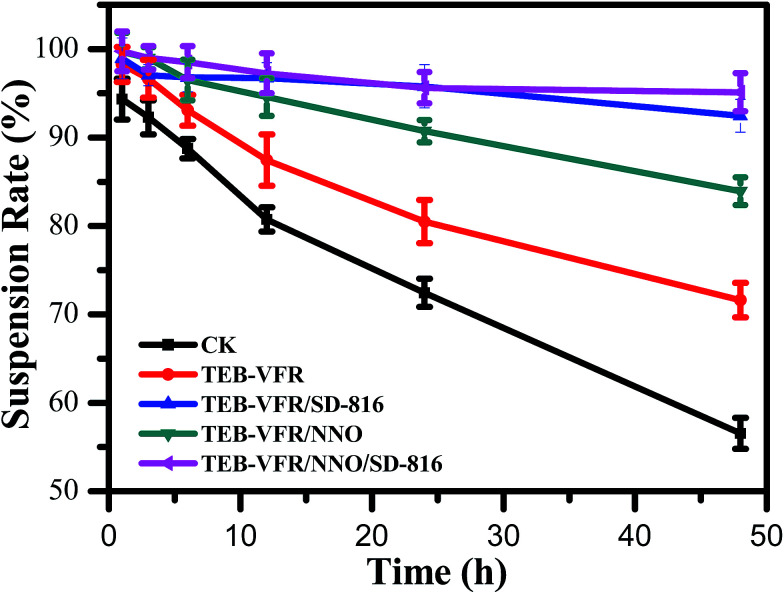
Suspension stabilities of the various TEB–VFR FS.

To characterize the stability performance of a series of self-made FS, we systematically investigated the suspension rate. Suspension rate is an important indicator of the physical stability of the FS, which is a comprehensive reflection of the particle size and the particle suspension performance in the system.^30,31^ As shown in Fig. 3, the suspension rate of CK system is only 56.6% after 48 h, however, the suspension rate of TEB–VFR increases to 71.6%, which may be attributed to the fact the VFR acts as effective carriers with small particle diameter (Fig. 1c) improving the suspending property of TEB–VFR system. Furthermore, for TEB–VFR/SD-816 and TEB–VFR/NNO systems, the suspension rates of the systems are significantly improved, and the suspension rates are 92.5% and 83.9%, respectively. This finding indicates that dispersant SD-816 and NNO play an important role and serve as effective dispersants for dynamically improving the stability of the suspension systems. In addition, when both SD-816 and NNO are added to the TEB–VFR system, the suspension stability of the TEB–VFR/NNO/SD-816 system further improves compared with TEB–VFR/SD-816 system. Consequently, the combination of the two dispersants had a synergistic effect on the increase of the suspension rate of the system and could improve the dispersibility of the system to some extent.

**Fig. 4:**
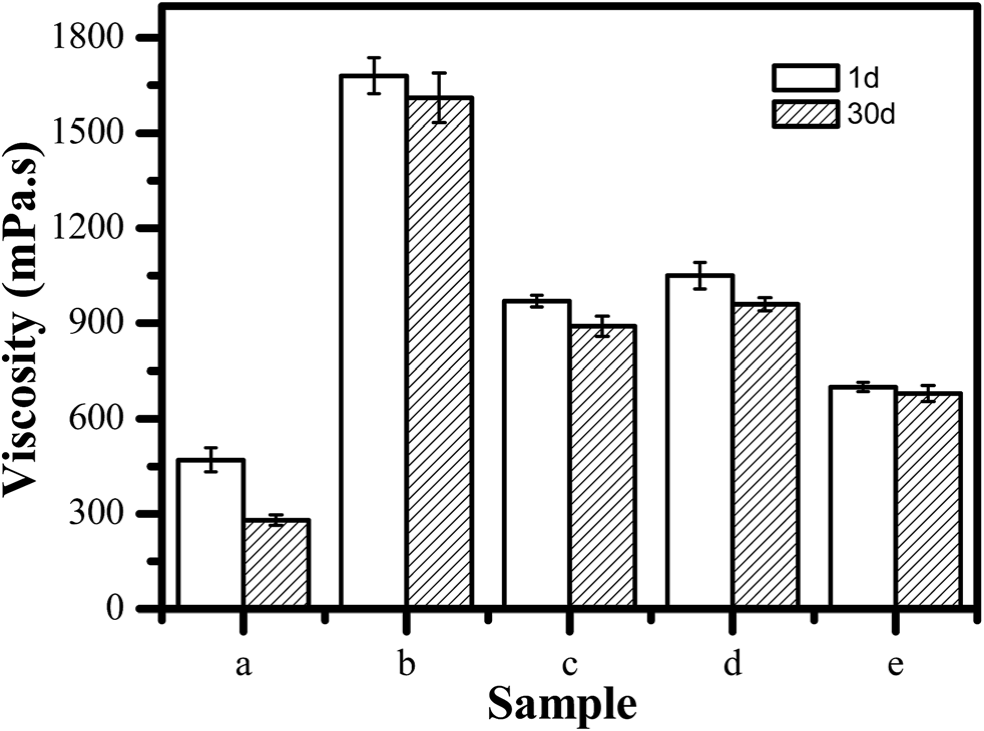
Viscosity change of the different TEB–VFR FS on the first and 30th day, (a) CK, (b) TEB–VFR, (c) TEB–VFR/NNO, (d) TEB–VFR/SD-816 and (e) TEB–VFR/SD-816/NNO.

The system viscosity has a significant influence on the stability of FS and the firmness of coating film.^32^ To further evaluate the suspension stability of each system, we assessed the viscosity changes of the various systems. Fig. 4 shows the viscosity of the various FS systems on the first and 30th day, respectively. For CK, the viscosity of CK has only 470 mPa s on the first day. Moreover, its viscosity exhibits significantly reduction on the 30th day. In contrast, for TEB–VFR FS system, compared to the CK, its viscosity increases dramatically. It has the maximum viscosity as high as 1680 mPa s. Furthermore, the TEB–VFR FS system exhibits more stable, and the viscosity change is small. The above results suggested that VFR can greatly enhance the viscosity of TEB–VFR FS, and facilitate the improvement of the system stability, which may be attributed to promotion agglomeration of particles, resulting in poor mobility and high viscosity of the TEB–VFR FS system.

To address the high viscosity of the TEB–VFR FS system, we attempted to add dispersant to TEB–VFR FS system to reduce the system viscosity, because dispersant is conducive to promote the dispersion of the TEB–VFR particles and prevents the flocculation and aggregation of the particles. As shown in Fig. 4c and d, both dispersant NNO and SD-816 significantly reduce the viscosity of the viscosities of TEB–VFR/NNO and TEB–VFR/SD-816 because of their superior dispersion of particles compared to TEB–VFR FS system. In addition, the viscosity changes of two systems are small, suggesting that both of TEB–VFR systems containing dispersant SD-816 or NNO had remarkable stability. However, the viscosity of FS should be less than 800 mPa s,^33^ TEB–VFR/NNO and TEB–VFR/SD-816 FS have higher viscosity. Thereby owing to their effective dispersion for TEB–VFR, we attempted to incorporate SD-816 and NNO into TEB–VFR FS, aiming to achieve the maximum level of viscosity improvement. Surprisingly, TEB–VFR/SD-816/NNO FS has proper viscosity, and achieves the requested viscosity. Moreover, its viscosity has only minor change on the first and 30th day, suggesting that the combination of two dispersants is beneficial to reduce the viscosity dispersant of TEB–VFR FS and improve dispersion stability of TEB–VFR. Therefore, TEB–VFR/SD-816/NNO FS was optimal choice. And its physicochemical properties are shown in ESI Table S1.†

**Fig. 5:**
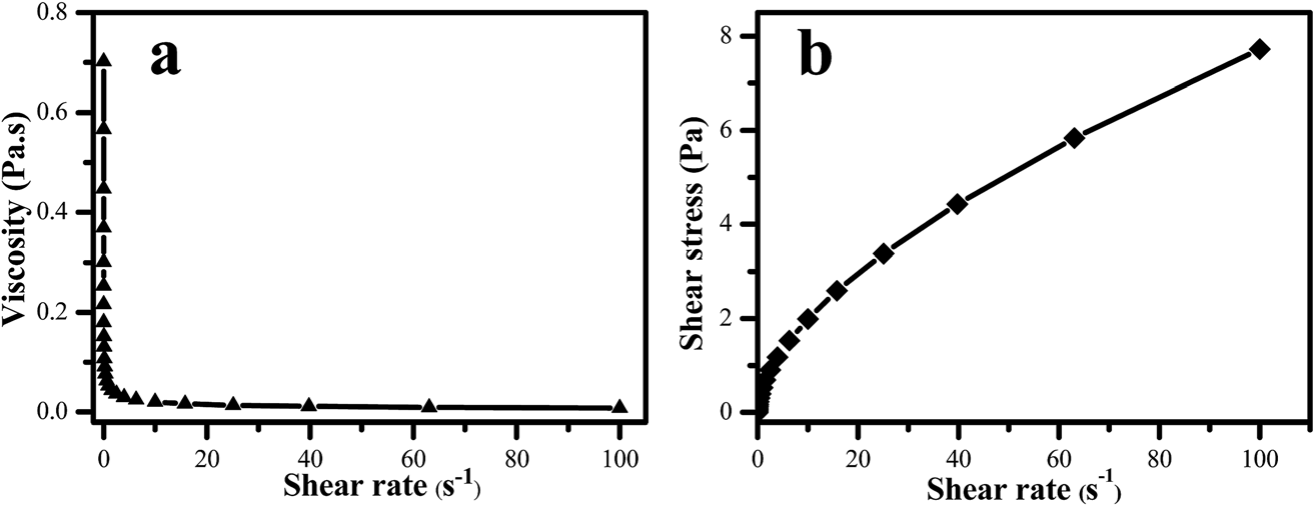
Rheological curve of TEB–VFR/SD-816/NNO FS.

The dispersion stabilities of FS systems were also investigated by rheological method.^34^ Moreover, the system rheology is the most critical technical parameters, it ensures that the FS can uniformly cover the seed surface, and it has a direct impact on the coating quality and efficacy. The rheological curve of TEB–VFR/SD-816/NNO FS is shown in Fig. 5. The apparent viscosity decreases with increasing shear rate (Fig. 5a), while the shear stress increases as the increase of shear rate (Fig. 5b). The results showed that the suspension system exhibited shear thinning behavior which is in accord with typical non-Newtonian pseudo-plastic fluid. That is, as the increase of shear rate, the directions and arrangements of the particles in the suspension system have changed, and the network structure has been destroyed, leading to decline of apparent viscosity. Moreover, when the shear rate is greater than 20 s^−1^, the network structure of the system is completely destructed, then the apparent viscosity remains unchanged, and the system shows the characteristic of shear thinning.^35^

The experimental data of the obtained FS were fitted according to the Herschel–Buckley formula.^35^*τ* = *τ*_H_ + *k*_H_*D*^*n*^where *τ* is shearing stress value (Pa), *τ*_H_ is yield value (Pa), which is indicative of strength of the system internal structures and suspension stability, the stability of the system is proportional to the yield value. *k*_H_ is viscosity coefficient, which shows the fluid mobilities, and the values of *k*_H_ is greater, the fluidity of fluid the poorer. *D* is shearing rate (s^−1^), *n* is flow-behavior index, *n* < l is a pseudo plastic fluid in non-Bingham fluids, *n* > l is an expansion fluid in non-Bingham fluids, and *n* = 1 is Bingham fluid.^35^ The fitting rheological parameters of TEB–VFR/SD-816/NNO FS and the other FS systems are shown in Table 1.

**Table 1:** Rheological parameter of different FS

Sample	*τ*_H_	*k*_H_	*n*	*R* ^2^
CK	0.0140	0.5157	0.5846	0.9964
TEB–VFR	2.2317	1.4432	0.4513	0.9971
TEB–VFR/SD-816	0.4196	1.1699	0.4926	0.9973
TEB–VFR/NNO	0.3374	0.8235	0.5183	0.9943
TEB–VFR/SD-816/NNO	0.4781	0.6329	0.5939	0.9975

According to Table 1, the fitting equations of various FS conform to the Herschel–Buckley model. The flow-behavior index *n* of different FS are less than 1, indicating that all kinds of FS show “shear thinning” pseudo-plastic characteristics. Compared to the other FS systems, the TEB–VFR and TEB–VFR/SD-816/NNO FS systems have higher yield value *τ*_H_, and exhibit excellent stabilities. Moreover, TEB–VFR has the highest *k*_H_, which indicates that the TEB–VFR FS possesses the largest viscosity and the worst mobility in line with Fig. 4. However, the *k*_H_ of TEB–VFR/SD-816/NNO is only 0.6329, suggesting it has good fluidity in accordance with its viscosity behavior (Fig. 4).

To evaluate the safety of TEB–VFR/SD-816/NNO FS, we assessed its effect on germination rate of wheat seeds, and tested for bacteriostatic activity against *Fusarium graminearum* and its protective effect against wheat powdery mildew. As shown in Table 2, wheat seeds coated by VFR/SD-816/NNO possess preferable germination rate compared to that of SD-816/NNO and CK. This shows that VRF can prove a beneficial effect on improving germination rate. The above results confirm our conjecture in Fig. 1. Additionally, wheat seeds coated with two FS show higher GP, GE, GI and VI compared to uncoated wheat seeds. It is noteworthy that the germination rate of wheat seeds coated with TEB–VFR/SD-816/NNO FS behave the most outstanding relative to the others.

**Table 2:** Effect of various FS on germination rate of wheat seeds^a^

Sample	GP (%)	GE (%)	GI (%)	VI (g)
CK	83.0 ± 2.0	0.53 ± 0.04	6.53 ± 0.1	32.60 ± 0.5
SD-816/NNO	83.4 ± 1.7	0.57 ± 0.06	6.57 ± 0.3	33.20 ± 0.4
VFR/SD-816/NNO	86.2 ± 2.1	0.63 ± 0.04	7.27 ± 0.3	41.20 ± 0.5
TEB–VFR/SD-816/NNO	90.0 ± 1.9	0.73 ± 0.03	8.74 ± 0.2	48.90 ± 0.4
Raxil	87.0 ± 1.5	0.60 ± 0.02	7.34 ± 0.1	37.40 ± 0.3

a GP: germination percentage, GE: germination energy, GI: germination index, and VI: vitality Index. The data are expressed as mean ± standard deviation. Means with different letters is significance different at *p* < 0.05.

Antibacterial experiment (Table 3) indicated that the FS systems of VFR/SD-816/NNO, SD-816/NNO and CK have no antibacterial activity. Furthermore, TEB–VFR/SD-816/NNO and Raxil FS possess superior antimicrobial activities. Additionally, compared to the commercial formulation, the TEB–VFR/SD-816/NNO FS exhibits excellent antibacterial efficacy against *Fusarium graminearum* and powdery mildew, particularly at a pharmacopoeia ratio of 1 : 50.

**Table 3:** Antibacterial test of TEB–VFR/SD-816/NNO FS^a^

Sample	CK	SD-816/NNO	VFR/SD-816/NNO	Raxil	TEB–VFR/SD-816/NNO		
					1 : 40	1 : 50	1 : 60
Fusarium graminearum	—	—	—	78.5 ± 2.1	73.8 ± 1.8	80.5 ± 2.3	77.9 ± 1.3
Powdery mildew	—	—	—	71.3 ± 1.4	67.5 ± 1.2	74.1 ± 2.1	70.9 ± 1.7

a Values are the mean ± SD of three replicates.

The bioactivities of the TEB–VFR/SD-816/NNO FS and commercial formulation of Raxil against wheat powdery mildew were conducted. As shown in Table 4, the results reveal that the control efficacies of two FS formulations are obviously superior to the control group. More strikingly, the TEB–VFR/SD-816/NNO FS exhibits excellent control efficacy against wheat powdery mildew in comparison with Raxil, and control efficacy of TEB–VFR/SD-816/NNO FS is 79.64 ± 1.92, but that of the commercial product is merely 75.32 ± 1.37. Obviously, the TEB–VFR/SD-816/NNO FS had remarkable advantages in providing protection effect on wheat.

**Table 4:** Emergence rate of wheat seeds after 7 days and control efficacy of TEB–VFR/SD-816/NNO FS and Raxil on wheat powdery mildew after 12 days^a^

Sample	Emergence rate (%)	Control Efficacy (%)
CK	93.3 ± 0.06	0
Raxil	100 ± 0	75.32 ± 1.37
TEB–VFR/SD-816/NNO	96.7 ± 0.06	79.64 ± 1.92

a Values are the mean ± SD of three replicates.

## Conclusions

In summary, we have successfully developed an efficient tebuconazole FS by using VFR as a potential pesticide platform. Furthermore, the obtained FS prepared by the VFR, dispersant SD-816 and NNO exhibits superior suspension stability. In addition, the system exhibits shear thinning behavior under high speed shearing, showing typical features of pseudo-plastic non-Newtonian fluids, which conforms to the Herschel–buckley model. Finally, the control efficacy of the optimal TEB–VFR/SD-816/NNO FS against wheat powdery mildew is superior to the control group and positive control Raxil, respectively. Accordingly, fermentation residues of validamycin are employed as a promising pesticide carrier for the fabrication FS.

## Conflicts of interest

There are no conflicts to declare.

## Supplementary Material

RA-008-C7RA12298E-s001
