# Supplementary material for: Utilization of the fermentation residues of validamycin as a potential platform for seed treatment
Source: RSC Adv. 2018 Mar 9;8(18):9956–62. doi: 10.1039/c7ra12298e (PMC9078719; doi:10.1039/c7ra12298e)
Supplement: RA-008-C7RA12298E-s001 [file RA-008-C7RA12298E-s001.pdf]

Electronic Supplementary Material (ESI) for RSC Advances.
This journal is © The Royal Society of Chemistry 2018

Supporting information

Utilization of fermentation residues of validamycin as a potential platform for seed treatment

Bo Zhang, Shaowei Liao and Tianrui Ren*

The Key Laboratory of Resource Chemistry of Ministry of Education, The Development Centre of Plant Germplasm Resources, College of Life and Environmental Science, Shanghai Normal University, 100 Guilin Road, Shanghai, 200234, P. R. China

* Corresponding author: Prof. Tianrui Ren

Phone: +86 21 64328850; Fax: +86 21 64328850

E-mail address: trren@shnu.edu.cn

Table S1. Physicochemical property analysis of TEB-VFR/SD-816/NNO FS

Sample	Suspension rate (%)	Viscosity (mPa.s)	pH	Film-forming effect	Coating uniformity (%)	Abscission rate (%)	Thermal stability (%)	Cold storage stability (%)
TEB-VFR/SD-816/NNO	96.4	670	6.26	Good	95	5.8	93.8	93.2
Standard[1,2]	>90	300-800	5 ~7	Good	>90	<10	>90	>90

References

1 Shenyang Research Institute of Chemical Industry. The methods of pesticide suspension: GB/T 14825-2006. Beijing: China Standard Press, 2006. (In Chinese)

2 Institute for the Control of Agrochemicals, Ministry of Agriculture. Guidelines on drafting standards of suspension concentrates for seed dressing: GB/T 17768-1999. Beijing: China Standard Press, 1999. (In Chinese)
